# House Appointments at Small Hospitals

**Published:** 1908-02-08

**Authors:** 


### Resident Medical Officers' Department.

### HOUSE APPOINTMENTS AT SMALL HOSPITALS.

There seems to be a little difficulty in getting good men to take up house surgeoncies at the smaller provincial hospitals, where there is only one resident. Yet these "one-man" hospitals afford experience of a more varied character than many large institutions, on account of the multifarious nature of the resident's duties. The remuneration given is usually good—about £100 per annum—and although there may be some truth in the saying "The less the pay, the better the experience," the reverse does not always hold good; for decidedly the experience to be gained at even a small hospital is eminently worth having, especially to a man who intends to enter general practice. The life is not too full of stress, the creature comforts are generally well looked after, and a considerable portion of social pleasures may come that way. These one-man hospitals consist of from 30 to 60, or even 70, beds, an out-patient attendance of three or four times a week, and often a special department for eyes or children. The staff is composed of some half-dozen of the best practitioners in the locality, and hence the work is bound to be a reflex of general practice. Thus the R.M.O. will have the advantage of studying their methods and of picking up many useful hints. In addition he will occasionally be taken out to assist at operations in private (usually as anæsthetist), and thereby cannot fail to learn much in the management of such cases, to say nothing of the addition to his income. As regards clinical work, he will find that he can afford time to go into his cases fairly fully, and can indulge any propensity he may have for hæmatology, etc. He can also find (or even make) opportunities for perfecting himself in the use of the laryngoscope and ophthalmoscope. The number of out-patients on any day will probably not be so excessive but that he can take an individual interest in a large number of them. He may acquire a useful facility in writing prescriptions, and will be less likely to get into the "hospital manner" than if he had a large crowd every day. The amount of dispensing the house surgeon will have to do varies in different institutions, and is yearly becoming less, as committees are recognising that it is a real economy to have a dispenser to attend daily. But, rightly used, this part of his work would afterwards stand him in good stead. In a small hospital the house surgeon comes into more intimate association with the nurses and sisters than he does in a London hospital, and for this reason he can learn incidentally many little details, the great importance of which in private practice he cannot fail to realise, and of which he learns nothing in his student days. In the majority of this class of appointments the house surgeon fills the *rôle* of anæsthetist; this experience is likely to be of more use to him in practice than a facility in performing laparotomies. Radiography is often part of his duty, and the knowledge thus acquired or perfected may help him to score later. Interviewing travellers may be made to serve a useful purpose, and in doing his best for his committee he will learn how to obtain the best value in instruments, dressings, etc. Lecturing to the nursing staff is a truly valuable training and a pleasant task. The casualty department will offer him many opportunities of proving himself, and not a few pitfalls also will offer in his path. At these times he has to act in the first instance unsupported, and it, therefore, is the most valuable part of his office. Lastly, his unique position in a small town is one of some importance, and, generally speaking, he is not so tied to the hospital as might at first sight appear.

